# Hepatic‐Accumulated Obeticholic Acid and Atorvastatin Self‐Assembled Nanocrystals Potentiate Ameliorative Effects in Treatment of Metabolic‐Associated Fatty Liver Disease

**DOI:** 10.1002/advs.202308866

**Published:** 2024-01-09

**Authors:** Huanfen Lu, Zhenglan Ban, Kai Xiao, Madi Sun, Yongbo Liu, Fangman Chen, Tongfei Shi, Li Chen, Dan Shao, Ming Zhang, Wei Li

**Affiliations:** ^1^ School of Biomedical Sciences and Engineering South China University of Technology Guangzhou Guangdong 511442 China; ^2^ National Engineering Research Center for Tissue Restoration and Reconstruction South China University of Technology Guangzhou Guangdong 510006 China; ^3^ College of Chinese Medicinal Materials Jilin Agricultural University Changchun 130118 China; ^4^ School of Medicine South China University of Technology Guangzhou Guangdong 510006 China; ^5^ College of Medicine Jilin University Changchun 130021 China

**Keywords:** atorvastatin, inflammation, metabolic‐associated fatty liver disease, nanocrystal, obeticholic acid

## Abstract

Exploration of medicines for efficient and safe management of metabolic‐associated fatty liver disease (MAFLD) remains a challenge. Obeticholic acid (OCA), a selective farnesoid X receptor agonist, has been reported to ameliorate injury and inflammation in various liver diseases. However, its clinical application is mainly limited by poor solubility, low bioavailability, and potential side effects. Herein a hepatic‐targeted nanodrugs composed of OCA and cholesterol‐lowering atorvastatin (AHT) with an ideal active pharmaceutical ingredient (API) content for orally combined treatment of MAFLD is created. Such carrier‐free nanocrystals (OCAHTs) are self‐assembled, not only improving the stability in gastroenteric environments but also achieving hepatic accumulation through the bile acid transporter‐mediated enterohepatic recycling process. Orally administrated OCAHT outperforms the simple combination of OCA and AHT in ameliorating of liver damage and inflammation in both acetaminophen‐challenged mice and high‐fat diet‐induced MAFLD mice with less systematic toxicity. Importantly, OCAHT exerts profoundly reverse effects on MAFLD‐associated molecular pathways, including impairing lipid metabolism, reducing inflammation, and enhancing the antioxidation response. This work not only provides a facile bile acid transporter‐based strategy for hepatic‐targeting drug delivery but also presents an efficient and safe full‐API nanocrystal with which to facilitate the practical translation of nanomedicines against MAFLD.

## Graphical abstract

A hepatic‐targeted nanodrug composed of obeticholic acid (OCA) and atorvastatin (AHT) is created to improve the stability in gastroenteric environments, while leveraging hepatic accumulation through apical sodium‐dependent bile acid transporter. Oral administration of the nanodrug outperforms the simple combination of OCA and AHT in ameliorating liver damage and inflammation in mice with acute liver injury or metabolic‐associated fatty liver disease.

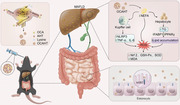

## 1 Introduction

Metabolic‐associated fatty liver disease (MAFLD), previously named nonalcoholic fatty liver disease (NAFLD), constitutes the most prevalent hepatic ailment, impacting a quarter of the global population.^[^ ^1^ ^]^ Patients with untreated MAFLD develop steatohepatitis with fibrosis, which can progress to more severe cirrhosis and carcinoma.^[^ ^2^ ^]^ Accumulating evidence from the epidemiology of MAFLD reveals that chronic excessive energy intake increases the accumulation of fat, leading to oxidative stress, lipid peroxidation, and chronic inflammation in hepatocytes.^[^ ^3^ ^]^ The current clinical management strategy for MAFLD includes lifestyle intervention, control of metabolic and modifiable risk factors, and prevention of hepatic complications.^[^ ^4^ ^]^ Nevertheless, none of the agents specifically targeting MAFLD have received approval from the Food and Drug Administration (FDA) due to their inability to meet the necessary clinical effectiveness and biosafety criteria.^[^ ^5^ ^]^ Therefore, there remains an urgent need to seek medicines for the efficient and safe management of MAFLD.

The farnesoid X receptor (FXR) is a nuclear receptor prominently expressed in the liver, regulating the metabolism of bile acid, cholesterol, and glucose, along with influencing hepatic inflammation and apoptosis.^[^ ^6^ ^]^ Obeticholic acid (OCA), a semisynthetic bile acid derivative, is approved by the FDA for primary biliary cholangitis through FXR activation.^[^ ^7^ ^]^ Specifically, OCA is emerging as a vital pharmacotherapy that not only ameliorates liver injury and inflammation but also improves clinical outcomes in MAFLD patients with different histological, metabolic, and biochemical issues.^[^ ^8^ ^]^ Nevertheless, higher dosing of OCA leads to elevated rates of pruritus and adverse alterations in the plasma lipid profile, including elevated low‐density lipoprotein‐cholesterol (LDL‐C) and reduced high‐density lipoprotein‐cholesterol (HDL‐C) levels.^[^ ^9^ ^]^ Statins are effective cholesterol‐lowering drugs widely used for managing or preventing both cardiovascular disease and MAFLD.^[^ ^10^ ^]^ A recent clinical trial evaluating the impact of statins with OCA therapy on the lipid profile of patients suffering from MAFLD revealed that increased LDL levels by OCA among patients were reduced using atorvastatin (AHT).^[^ ^11^ ^]^

Despite the pharmacological benefits of combining OCA and AHT in MAFLD management, these combos suffer from poor solubility and severe side effects during oral administration, thus severely restricting their clinical applications.^[^ ^12^ ^]^ In recent years, there has been substantial research interest in utilizing nanocrystal‐based delivery systems as a versatile approach for poorly soluble drugs.^[^ ^13^ ^]^ Compared to free drugs, nanocrystals have prolonged bioavailability, enhanced cellular penetration, and specific organ accumulation.^[^ ^14^ ^]^ Beyond these advantages, carrier‐free nanocrystals overcome the challenges posed by both the low loading content of active pharmaceutical ingredients (APIs) and the potential toxicity linked to excipients in nanomedicine.^[^ ^15^ ^]^ Given the enterohepatic recycling process of bile acids, utilizing bile acid transporter‐mediated oral drug delivery has been acknowledged as a practical and promising strategy for enhancing oral bioavailability.^[^ ^16^ ^]^ Considering these findings, we postulate that OCA (a bile acid derivative) might be considered a promising candidate for bile acid transporter‐mediated oral drug delivery, which has not been reported yet.

In this study, we create a carrier‐free full‐API nanocrystal (OCAHT) based on clinically approved OCA and AHT for oral combination therapy of MAFLD. On the one hand, the formation of nanoparticulate OCAHTs is dominantly driven by weak noncovalent forces, including hydrogen bonds, van der Waals forces, and hydrophobic interactions. Attractively, such multiple interactions enhance their stability in gastroenteric environments, while the presence of OCA endows these nanocrystals with hepatic accumulation capacity through the enterohepatic recycling process of bile acid mimetics. On the other hand, OCAHT effectively reverses MAFLD with fewer side effects by ameliorating liver injury and inflammation. OCAHT, featuring a commendable API content, precise liver targeting, and the incorporation of synergistic therapeutics, holds the potential to broaden the horizons of efficient and secure nanomedicine development for the management of MAFLD.

## 2 Results and Discussion

### 2.1 Preparation and Characterization of OCAHT

**Figure 1:**
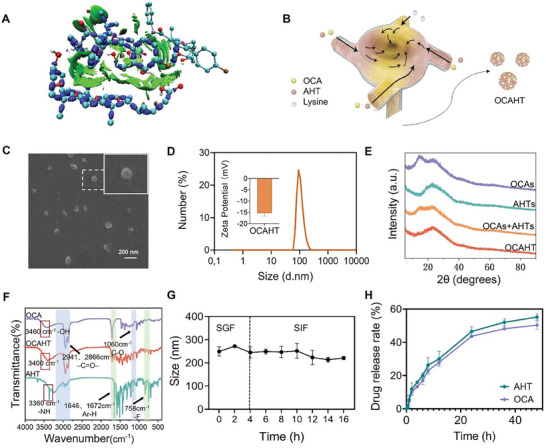
Preparation and characterization of OCAHT nanocrystals. A) 3D iso‐surfaces of OCA and AHT. B) Schematic illustration of the preparation procedure of OCAHT nanocrystals. C) The morphology of OCAHT was characterized by SEM. Scale bar: 200 nm. D) Size distribution and zeta potential of OCAHT were determined by dynamic light scattering (DLS). E) XRD of OCAHT nanocrystals and other formulations. F) FTIR spectra of OCA, AHT, and OCAHT. G) Stability of OCAHT in SGF and SIF. H) Drug‐release profiles of OCAHT in SBF.

To assess the feasibility of self‐assembling OCA and AHT, molecular docking was performed to predict the interaction between the two drug molecules prior to material preparation. The binding energy between OCA and AHT was −4.5 kJ mol^−1^ (Figure S1, Supporting Information), indicating the possibility of the self‐assembly of OCA and AHT.^[^ ^17^ ^]^ Beyond hydrophobic interactions, the hydrogen bond and van der Waals force determined by the interaction region indicator (IRI) assay contributed to driving the interaction between OCA and AHT (**Figure** **1**A; Figure S2, Supporting Information). As illustrated in Figure 1B, carrier‐free OCAHTs were prepared according to a facile and versatile antisolvent strategy, in which self‐assembly was facilitated in a multiple inlet vortex mixer (MIVM) through scalable and repeatable flash nanocomplexation (FNC).^[^ ^18^ ^]^ OCAHT nanocrystals were uniformly spherical, possessing an average diameter of ≈130 nm and a negative charge (−15.3 ± 0.78 mV) (Figure 1C,D). The difference in the X‐ray diffraction (XRD) parameters revealed that OCAHT, as an amorphous nanocrystal, was formed via instantaneous assembly of OCA and AHT (Figure 1E; Figure S3, Supporting Information). The Fourier‐transform infrared (FTIR) spectra of OCAHT exhibited the classic peaks of ─C═O─ tensile vibration at 2941 and 2866 cm^−1^ and C─O tensile vibration at 1060 cm^−1^ (Figure 1F), suggesting the preservation of OCA. Similarly, the characteristic aromatic hydrogen and fluorine peaks of AHT located at 1646, 1572, and 758 cm^−1^ could also be found in OCAHT. Furthermore, the FTIR analysis proved the presence of intermolecular coordination bonds and hydrogen bonds owing to the carboxyl group of OCA (3460 cm^−1^) and the calcium ion and amide group of AHT (3360 cm^−1^), which might facilitate the instantaneous assembly (Figure 1F).

Through high‐performance liquid chromatography (HPLC) and UV analysis, the individual drug loading percentages of OCA and AHT were determined as 37.42% and 48.08%, respectively. The OCAHT nanocrystals exhibited a total drug loading capacity close to 90%. Such an ultrahigh API content might reduce the toxic concerns raised by synthetic carriers. Given that the dissolution rate and dispersion stability of poorly water‐soluble molecules could be improved after nanocrystallization, we investigated the stability and drug release profile of OCAHT nanocrystals for oral delivery.^[^ ^19^ ^]^ To explore the stability of OCAHT in the harsh conditions of the gastrointestinal tract, OCAHT underwent 4 h incubation with simulated gastric fluid (SGF), followed by a subsequent 12 h exposure to simulated intestinal fluid (SIF). The size of OCAHT nanocrystals changed slightly in SIF and SGF (Figure 1G). The transmission electron microscopic (TEM) images also confirm that OCAHTs could largely maintain their morphology after incubation with gastroenteric fluid (Figure S4, Supporting Information). We studied the release profile of OCAHTs in simulated body fluid (SBF) (Figure 1H). The OCAHT nanocrystals demonstrated sustained release profiles, with OCA and AHT showing 50.31% and 54.64% release at 48 h, respectively, which were slower than the release of free OCA or AHT (Figure S5, Supporting Information). These findings suggest that OCAHT nanocrystals may coordinate the release of OCA and AHT under physiological conditions. Taken together, these findings demonstrate that our OCAHT nanocrystals exhibit a high API content, good stability, and a sustained‐release manner, which might be suitable for oral administration in liver disease treatment.

### 2.2 OCAHT Mainly Accumulated in the Liver After Oral Administration

**Figure 2:**
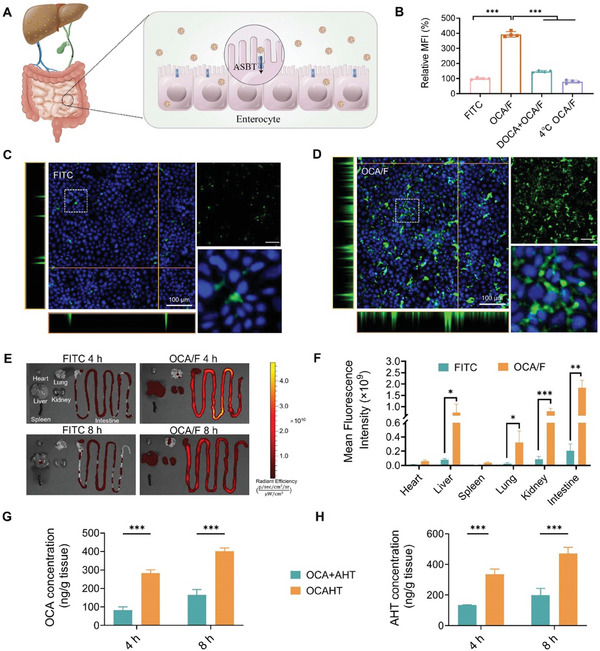
The cellular internalization and hepatic accumulation of OCAHTs are much higher than that of free drugs. A) Schematic illustration of how orally administrated OCAHTs accumulate in the liver through ASBT‐mediated endocytosis for enterohepatic circulation. B) Quantitative detection of cellular internalization in various groups. Data are expressed as mean ± SD (*n* = 4), ^***^ *p* < 0.001. C,D) CLSM images of Caco‐2 cell monolayers incubated with FITC and OCA/F. Nuclei were stained with DAPI (blue). Scale bar: 100 µm. E) Fluorescence images of the main organs at 4 and 8 h after mice orally received FITC or OCA/F. F) Mean fluorescence intensity of organs shown in E at 8 h. G,H) Hepatic concentrations of OCA G) and AHT H) after oral administration of OCA + AHT or OCAHT for 4 and 8 h at OCA and AHT doses of 9.4 and 12 mg kg^−1^, respectively. Data are expressed as mean ± SD (*n* = 3), ^*^ *p* < 0.05, ^**^ *p* < 0.01, ^***^ *p* < 0.001.

Enterocytes primarily absorb bile acids via apical sodium‐dependent bile acid transporter (ASBT) and subsequently transport them from the intestine to the liver, thereby facilitating enterohepatic recycling.^[^ ^20^ ^]^ Given that OCA is a derivative of bile acid, we speculated that OCA‐based nanocrystals might be taken up by intestinal cells through ASBT‐mediated processes, leading to their specific accumulation in the liver (**Figure** **2**A). To test our hypothesis, we employed ASBT‐expressing Caco‐2 cells to study the uptake of OCAHT. To visualize the uptake of these nanocrystals, fluorescein isothiocyanate (FITC) was co‐assembled with OCA to form trackable nanocrystals (OCA/F) with similar size (≈150 nm) and zeta potential (−15.15 ± 2.55 mV) to OCAHTs (Figure S6, Supporting Information). The amounts of OCA/F across the Caco‐2 monolayer model were determined via flow cytometry analysis (FACS). The cellular fluorescence intensity of the OCA/F group was 3.92 times higher than that of the free FITC group (Figure 2B), while a 64.2% reduction in FITC uptake was found after pretreatment with sodium deoxycholate (DOCA), a competitor that blocks the interaction between OCA and ASBT, indicating that the cellular uptake of OCAHTs was partly attributed to the ASBT‐mediated endocytosis pathway. The cellular uptake study of OCA/F conducted at 4 °C revealed a significant decrease in nanocrystal endocytosis to 20.3% when compared to the uptake at 37 °C, suggesting a reliance on energy for cellular internalization. Next, the internalization of nanocrystals by the Caco‐2 monolayer was observed via confocal laser scanning microscopy (CLSM). Consistent with the quantitative results, OCA/F exhibited stronger green fluorescence than free FITC (Figure 2C,D; Figure S7, Supporting Information). The validity of this finding was additionally corroborated through the visualization of cell monolayers using the xz and yz images. (Figure 2C,D). FITC‐labeled OCAHT nanocrystals (OCAHT‐FITC) were further fabricated to confirm the advantages of OCAHT for cellular internalization (Figure S8, Supporting Information).

Having determined the ASBT‐mediated endocytosis of OCA/F, we sought to investigate its biodistribution through an in vivo imaging system (IVIS). After oral administration of OCA/F, the main organs of the experimental mice, including the heart, liver, spleen, lung, kidney, and intestine, were removed for ex vivo imaging at 4 and 8 h (Figure 2E,F; Figure S9, Supporting Information). The vast majority of OCA/F were trapped in the liver, kidney, and intestine. As expected, OCA/F exhibited higher accumulation in the liver than free FITC (Figure 2F). Consistently, OCAHT‐FITC exhibited preferable accumulation in the liver (Figure S10, Supporting Information). To further determine the biodistribution of OCAHT nanocrystals, OCA and AHT levels in mouse liver were quantified via HPLC. We found that both hepatic OCA and AHT levels were significantly higher in the OCAHT group when compared to the OCA + AHT group, which were similar to the fluorescent results of OCA/F or OCAHT‐FITC, suggesting that OCAHT might effectively facilitate the high accumulation of OCA and AHT in the liver (Figure 2G,H). Collectively, our findings indicated that OCA moieties might offer promising advantages toward the preferable hepatic accumulation of OCA‐based nanocrystals.

### 2.3 OCAHT Attenuated Liver Damage and Inflammation in Acetaminophen‐Challenged Mice

**Figure 3:**
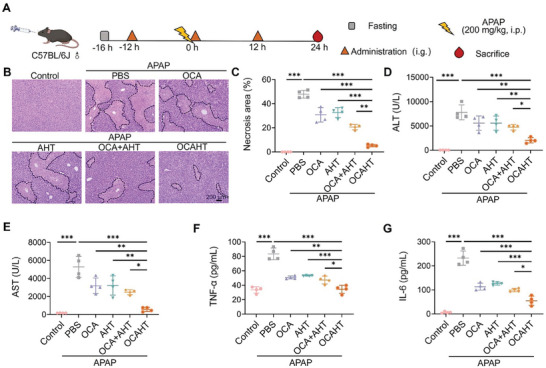
OCAHT protects against acute liver damage and inflammation. A) Schematic illustration of the development of APAP‐challenged mice and the therapeutic effect of OCAHT against ALI. B) Representative H&E staining of the liver. Scale bar: 200 µm. C) The level of necrosis area. D–G) The levels of ALT, AST, TNF‐α, and IL‐6 in serum at 24 h after APAP challenged. Data are expressed as mean ± SD (*n* = 4), ^*^ *p* < 0.05, ^**^ *p* < 0.01, ^***^ *p* < 0.001.

Acetaminophen (APAP) overdose leads to severe acute liver injury (ALI) by inducing hepatocyte damage and inflammatory response.^[^ ^21^ ^]^ OCA inhibits acute liver injury by activating FXR, while the anti‐inflammatory effects of statins have been reported in both animal and clinical studies.^[^ ^10^, ^22^ ^]^ Based on these findings, we sought to investigate the protective effects of OCAHT on APAP‐challenged mice. An acute liver injury mouse model was induced by administering a high dose (200 mg kg^−1^) of APAP via intraperitoneal injection. OCA, AHT, OCA + AHT, or OCAHT (OCA: 9.4 mg kg^−1^, AHT: 12 mg kg^−1^) was orally administered three times (at 12 h before APAP challenge, and 1 and 12 h after APAP challenge) (**Figure** **3**A). Liver damage was assessed by quantifying the area of necrosis in the liver sections stained with H&E (Figure 3B,C). The APAP‐challenged mice exhibited significant hepatic cell necrosis and disruption of hepatocyte architecture compared to the control mice (Figure 3B). Our findings indicate that both single therapies, OCA or AHT, and the combination therapy of OCA + AHT significantly reduced the quantitative area of liver necrosis (Figure 3C). Amazingly, the necrotic area of the APAP‐challenged mice decreased from 47.86 ± 3.24% to 5.23 ± 1.07% after treatment with OCAHT. In addition to the necrotic area, serum alanine aminotransferase (ALT) and aspartate aminotransferase (AST) levels are considered important indicators in clinical practice to diagnose ALI.^[^ ^23^ ^]^ In line with the findings of the quantitative necrosis area, both ALT and AST levels in the serum of APAP‐challenged mice were markedly elevated (Figure 3D,E). In contrast, combination therapy with OCA and AHT significantly reduced serum AST and ALT levels. Importantly, the OCAHT group rendered lower serum ALT and AST levels than the OCA + AHT group. Moreover, we investigated the therapeutic profile of mice receiving treatment with OCAHT at 1 and 12 h after the APAP challenge. Consistently, OCAHT outperformed OCA + AHT in the amelioration of the necrosis area, ALT, and AST levels in APAP‐challenged mice (Figure S11, Supporting Information). These findings collectively demonstrated the prophylactic and therapeutic advantages of OCAHT in APAP‐challenged mice.

To study the effect of OCAHT on inflammation, serum levels of proinflammatory cytokines, including tumor necrosis factor‐α (TNF‐α) and interleukin‐6 (IL‐6), were measured in each group. As expected, the serum levels of TNF‐α and IL‐6 of APAP‐challenged mice were found to be significantly increased in comparison to those obtained from the control group (Figure 3F,G). The liver damage conferred by OCA + AHT corresponded with a significant reduction in the serum levels of both TNF‐α and IL‐6. OCAHT exhibited the best anti‐inflammatory effects among all treatments. Taken together, the findings of this study demonstrated that OCAHT was more effective than the combination of OCA and AHT in reducing liver damage and inflammation in mice challenged with acetaminophen. To investigate the potential safety profile of OCA and AHT, healthy mice were subjected to repeated oral administration of OCA at a dose of 9.4 mg kg^−1^, along with AHT at a dose of 12 mg kg^−1^, over a duration of one week. There was no significant change in ALT and AST levels or liver histopathology in each group (Figure S12, Supporting Information), suggesting that OCA, AHT, and OCAHT exhibited negligible impacts on liver function at low dosages.

### 2.4 OCAHT Counteracted High‐Fat Diet‐Induced Liver Steatosis and Inflammation

**Figure 4:**
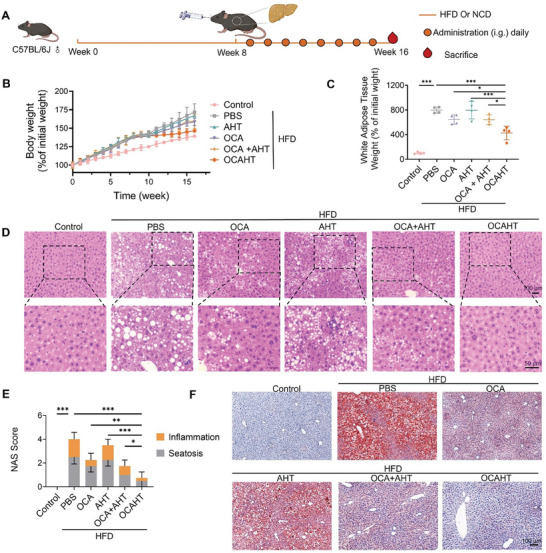
OCAHT ameliorates chronic liver steatosis and inflammation. A) Schematic illustration of the development of HFD‐induced mice and the therapeutic effect of OCAHT against MAFLD. B) Body weight of mice. C) White adipose tissue weight of mice after treatment. D) Representative H&E staining of the liver. Scale bar: 100 or 50 µm. E) NAS score of mice after treatment. F) Representative Oil Red O staining of the liver. Scale bar: 100 µm. Data are expressed as mean ± SD (*n* = 4), ^*^ *p*< 0.05, ^**^ *p* < 0.01, ^***^ *p* < 0.001.

Given the excellent therapeutic performance and reduced side effects of the combination of OCA and AHT for clinical MAFLD management, we speculated that OCAHT nanocrystals might promote the efficacy and safety of the combined treatment. We established a mouse model with mice receiving 8 weeks of a high‐fat diet (HFD), which is one of the most frequently employed models for studying MAFLD. Mice fed an HFD suffer from abnormal lipid metabolism, which leads to lipid accumulation, oxidative stress, and inflammation in the liver, mimicking the symptoms of human MAFLD.^[^ ^24^ ^]^ After 8 weeks of HFD challenge, the mice were orally administered OCA, AHT, OCA + AHT, or OCAHT every day at doses of OCA (9.4 mg kg^−1^) and AHT (12 mg kg^−1^) for another 8 weeks (**Figure** **4**A). The body weight of each mouse was monitored once a week (Figure 4B). As MAFLD is closely associated with obesity, over the 16‐week dosage period, the body weight of HFD‐challenged mice steadily increased. This resulted in an endpoint body weight of 37.43 ± 2.44 g, which was comparable to a weight gain of 71.67 ± 22.38% from the start of dosing. Administration of either OCA or OCA + AHT resulted in a decrease in the endpoint body weight compared to the MAFLD mice, while AHT had no obvious impact on weight gain. Importantly, OCAHT inhibited further weight gain (31.80 ± 0.39 g, 5.82 ± 1.30%) compared to the combination of OCA and AHT (34.05 ± 0.74 g, 13.50 ± 2.48%). Correspondingly, OCAHT induced a higher loss of fat weight than that of the combined therapeutics (Figure 4C), providing more evidence linking MAFLD to obesity. In line with these findings, OCAHT significantly reduced hepatomegaly in HFD‐challenged mice (Figure S13, Supporting Information).

Liver histopathology is considered the gold standard for diagnosing MAFLD.^[^ ^25^ ^]^ To precisely assess the impact of OCAHT on the liver pathology of MAFLD, the liver of the mice was collected at the end of the experiment, and the left outer lobe was chosen for hematoxylin‐eosin (H&E) staining (Figure 4D). In contrast to the control group, fat vacuolated of various sizes were found in the hepatocytes with loose cytoplasm obtained from HFD‐challenged mice, indicating apparent hepatocytic steatosis. The AHT group exhibited a degree of hepatocytic steatosis and inflammatory infiltration that ranked second only to the model mice. Both the OCA and OCA + AHT groups exhibited a positive therapeutic outcome by markedly improving fatty liver steatosis and inflammatory infiltration. The administration of OCAHT yielded the most significant reductions in the NAFLD activity score (NAS), primarily attributed to markedly decreased steatosis and inflammation scores (Figure 4E). Additionally, OCAHT led to comparable decreases in serum AST levels (Figure S14, Supporting Information). In order to distinctly identify lipid droplet‐containing cells and quantify their abundance, the liver samples were stained with oil red O. The obtained results exhibited similarities to those of H&E staining. Oil red O staining demonstrated a dramatic reduction in hepatic lipid droplet accumulation following the administration of OCAHT (Figure 4F), which was more efficient than the simple combination of OCA and AHT. Collectively, these results demonstrated that oral administration of OCAHT may offer potential benefits in ameliorating the pathological characteristics of MAFLD.

**Figure 5:**
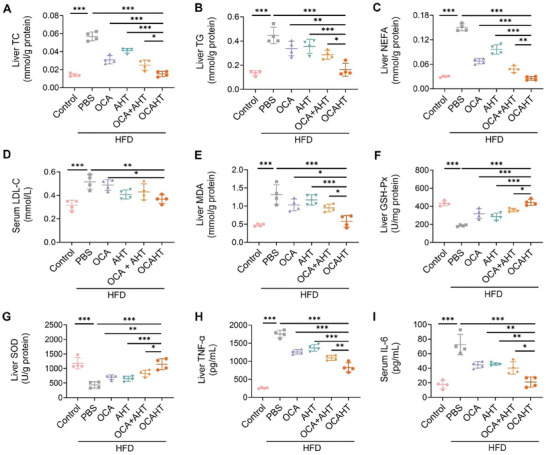
OCAHT reverses lipid metabolism, oxidative stress, and inflammation in MAFLD. A–C) The levels of TC, TG, and NEFA in the liver after treatment. D) The level of LDL‐C in serum after treatment. E–H) The levels of MDA, GSH‐Px, SOD, and TNF‐α in the liver after treatment. I) The level of IL‐6 in serum after treatment. Data are expressed as mean ± SD (*n* = 4), ^*^p< 0.05, ^**^ *p* < 0.01, ^***^ *p* < 0.001.

Lipids in the liver of patients with MAFLD mainly accumulate in the form of triglycerides (TG), which are mainly derived from the esterification of non‐esterified fatty acid (NEFA) and glycerol.^[^ ^26^ ^]^ Moreover, MAFLD has been associated with extensive dysregulation of hepatic cholesterol homeostasis, which results in elevated total cholesterol (TC) levels.^[^ ^27^ ^]^ We thus determined the levels of TC, TG, and NEFA in the liver of each mouse (**Figure** **5**A–C). A high‐fat diet led to substantial quantities of TC, TG, and NEFA, while OCAHT significantly dropped the levels of each biomarker regarding lipid accumulation in HFD‐challenged mice. Although OCA did not elevate serum LDL‐C levels, the combination of OCA and AHT reduced serum LDL‐C levels (Figure 5D). It is worth noting that OCAHT led to the most significant decline in both serum LDL‐C and TC levels in HFD‐challenged mice (Figure 5D; Figure S15, Supporting Information). Since the imbalance of redox homeostasis in the liver plays a critical role in the development and progression of MAFLD.^[^ ^28^ ^]^ We examined the hepatic levels of malondialdehyde (MDA), a byproduct of lipid peroxidation, and the activity of glutathione peroxidase (GSH‐Px) and superoxide dismutase (SOD) (Figure 5E–G). In HFD‐challenged mice, MDA levels dramatically increased, whereas GSH‐Px and SOD activities substantially decreased in the liver. The imbalance of redox homeostasis was partly reversed by the single or combined treatment. Importantly, OCAHT outperformed the other groups in increasing hepatic GSH‐Px and SOD activities and reducing MDA levels, thus improving the antioxidant capacity of the liver. We further investigated the effects of OCAHT on free fatty acid (FFA)‐induced hepatocyte damage in vitro. OCA and OCAHT exhibited significant inhibitory activity on lipid accumulation and oxidative stress (Figure S16, Supporting Information). The fluorescence of cellular lipids and reactive oxygen species (ROS) were significantly reduced by the OCAHT treatment when compared to the FFA‐challenged and OCA + AHT group. These results indicated that OCAHT might regulate lipid and redox homeostasis in hepatocytes and alleviate hepatocyte damage caused by FFA.

Inflammation also influences the pathogenesis of MAFLD, which is considered a driving force to promote liver fibrosis.^[^ ^2a^ ^]^ In addition to its lipid‐lowering effect, AHT also exhibits an anti‐inflammatory role, which is also reflected in the treatment of MAFLD.^[^ ^29^ ^]^ AHT decreased hepatic TNF‐α levels and serum levels of IL‐6 and TNF‐α in the MAFLD mice and exhibited a comparable anti‐inflammatory effect to OCA. Furthermore, the combination of these two drugs enhanced the anti‐inflammatory effect (Figure 5H,I; Figure S17, Supporting Information). However, OCAHT consistently reduced these pro‐inflammatory factors when compared to the other groups. According to these findings, OCAHT exhibited the most effective impact against chronic inflammation in MAFLD. To decipher the potent role of OCA and AHT in OCAHT‐induced effectiveness toward hepatic protection and anti‐inflammation, we next examined the effect of OCAHT‐induced lipopolysaccharide (LPS)‐activated proinflammatory macrophages since macrophages are major contributors to the inflammatory response in MAFLD.^[^ ^30^ ^]^ As expected, the level of the proinflammatory cytokine TNF‐α was significantly increased post‐LPS treatment. Both OCA and AHT demonstrated noticeable inhibitory effects on inflammation, while the OCAHT intervention exhibited a greater suppression of the elevation of TNF‐α than the combination of OCA and AHT (Figure S18, Supporting Information). Aligning with previous in vivo observations, we believe that OCAHT displays both hepatic protection and anti‐inflammatory effects. In such a scenario, its hepatic protective effect is mainly attributed to OCA, while its anti‐inflammatory effect is due to both OCA and AHT.

Finally, we evaluated the biocompatibility of OCAHT via H&E staining at the end of treatment (Figure S19, Supporting Information). Compared to the normal mice, no histopathological abnormalities caused by OCAHT were observed in the heart, spleen, lung, and kidney. Collectively, OCAHT exhibited a synergistic therapeutic effect that exceeded the combined effect of OCA and AHT, effectively mitigating the liver steatosis and inflammation induced by a high‐fat diet while minimizing systemic toxicity.

### 2.5 OCAHT Exerted Reverse Effects on MAFLD‐Associated Molecular Pathways

**Figure 6:**
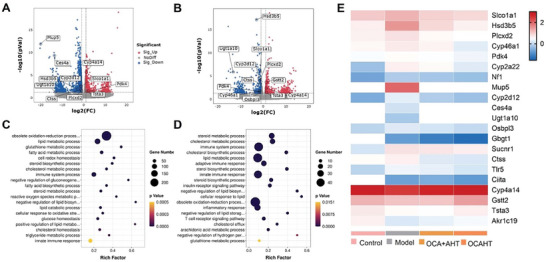
OCAHT treats MAFLD by regulating the expression of genes associated with lipid accumulation, inflammation, and oxidative stress. A,B) Volcano map of DEGs in mouse liver between OCA + AHT versus HFD A) and OCAHT versus HFD B) after treatment. C,D) GO functional enrichment analysis of DEGs between OCA + AHT versus HFD C) and OCAHT versus HFD D) after treatment. E) Heatmaps of significant DEGs involved in lipid metabolism, oxidative stress, and inflammation.

To gain a deeper understanding of the protective mechanisms of OCAHT against MAFLD, we performed pairwise analyses of liver tissues in the control, HFD, OCA + AHT, and OCAHT groups via RNA sequencing (RNA‐seq).^[^ ^31^ ^]^ The volcano plot analysis of differentially expressed genes (DEGs) revealed significant alterations in gene expression patterns across multiple comparisons: HFD versus control, OCA + AHT versus HFD, OCAHT versus HFD, and OCAHT versus OCA + AHT (**Figure** **6**A,B; Figure S20A,C, Supporting Information). The Gene Ontology (GO) enrichment analysis revealed that the HFD model exhibited significant activation of lipid accumulation, inflammatory response, and oxidative imbalance, all of which contributed significantly to the development of MAFLD (Figure 6C,D; Figure S20B,D, Supporting Information). The DEGs of the OCAHT group were substantially similar to those regulated by OCA + AHT, resulting in a gene expression pattern comparable to that of the HFD model (Figure 6C,D). Nevertheless, lipid metabolism and anti‐inflammatory effects might be associated with OCAHT being superior to the simple combination of OCA and AHT. To preliminarily identify the DEGs, we investigated candidate genes associated with MAFLD. The heatmap‐based representation revealed distinct gene expression pattern regulations in the OCAHT group when compared to the OCA + AHT group (Figure 6E). OCAHT suppressed MAFLD diet‐induced lipid accumulation gene expression (Hsd3b5, Plcxd2, Cyp2d12, Gbgt1, and PDK4). Notably, OCAHT attenuated MAFLD diet‐induced downregulation of key proinflammatory genes (Ctss, Tlr5, and Ciita) and upregulation of pivotal antioxidant genes (Cyp4a14, Tsta3, and Gstt2). Taken together, these findings revealed that OCAHT was more efficient in reversing impaired lipid metabolism, reducing inflammation, and enhancing the antioxidation response in comparison to the combined therapeutics during the treatment of MAFLD.

**Figure 7:**
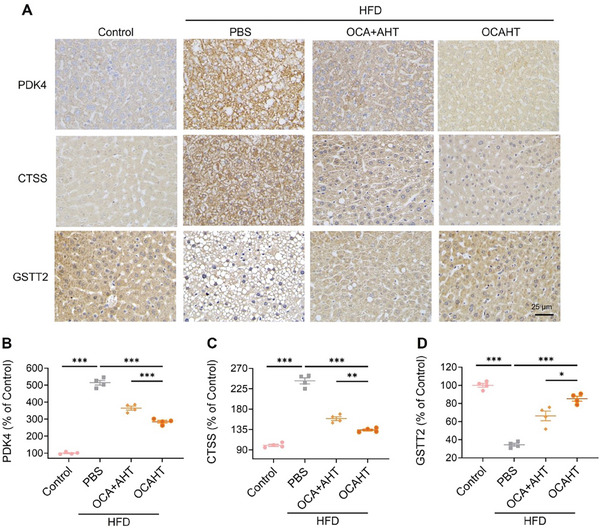
OCAHT orchestrates the expression of MAFLD‐related proteins. A) Representative immunohistochemical staining of the liver for PKD4, CTSS, and GSTT2 after treatment. Scale bar: 25 µm. B–D) Quantitative analysis results of in the levels of PKD4 B), CTSS C), and GSTT2 D) for images shown in A via ImageJ. Data are expressed as mean ± SD (*n* = 4), ^*^ *p* < 0.05, ^**^ *p* < 0.01, ^***^ *p* < 0.001.

According to the transcriptomic data, three differentially expressed genes were chosen: PDK4 (a lipid metabolism‐related protein), CTSS (an inflammation‐related protein), and GSTT2 (an antioxidant‐related protein) were selected for protein‐level immunohistochemistry (IHC) validation. The IHC results showed that the hepatic expression levels of the lipid metabolism‐related proteins PDK4, fatty acid binding protein 1 (FABP1), and peroxisome proliferator‐activated receptor γ (PPARγ) and the inflammation‐related proteins CTSS and NLRP3 were significantly elevated in HFD‐challenged mice (**Figure** **7**; Figure S21, Supporting Information). However, treatment with OCAHT effectively reversed their expression levels, demonstrating an enhanced effect compared to the OCA + AHT group. Moreover, the expression of the antioxidant‐related proteins GSTT2 and NF‐E2‐related factor 2 (Nrf2) was greatly increased in the livers of OCAHT‐treated mice compared to the HFD‐challenged mice. Aligning with the transcriptomic sequencing data, these findings demonstrated that OCAHT exerted profoundly reversed effects on MAFLD‐associated molecular pathways, such as lipid accumulation, inflammation, and oxidative stress.

## 3 Conclusion

In this study, we fabricated a nanodrug composed entirely of FDA‐approved OCA and AHT via self‐assembly for hepatic‐targeting treatment of MAFLD. By harnessing the collective interplay of diverse weak noncovalent forces, the resulting OCAHT exhibited heightened stability under physiological conditions and enhanced hepatic accumulation via the enterohepatic recycling process mediated by bile acid transporters. Importantly, oral administration of OCAHT potentiated the ameliorative effects of the OCA and AHT combination in both acetaminophen‐challenged mice and high‐fat diet‐induced MAFLD mice with negligible systemic toxicity. Specifically, OCAHT led to superior results in lipid accumulation, inflammation, and oxidative stress when compared with the simple combination therapy.

Given that utilizing bile acid transporter‐mediated oral drug delivery is considered a viable and promising approach to enhancing oral bioavailability, we first employed OCA, a bile acid mimetic, to achieve bile acid transporter‐mediated hepatic accumulation through the enterohepatic recycling process. In such a scenario, other APIs are also welcome to replace AHT, forming OCA‐based nanocrystals with increased effects during the combined treatment of liver diseases. With its nearly 100 wt% API content, improved colloidal stability, and prevention of premature release, the oral administration of OCAHT might effectively promote the protective effects of the combination of OCA and AHT and improve biosafety. Given these considerations, such simple and low‐cost nanocrystals based on OCA and other FDA‐approved drugs have the potential to not only demonstrate high‐performance efficacy through hepatic‐targeted delivery but also exhibit substantial promise for enhancing patient compliance in clinical applications. Despite further optimization that could be carried out, this OCA‐based nanodrug with hepatic‐targeting properties presents a promising strategy for expanding the horizons of synergistic therapy. Moreover, it will aid in the practical translation of OCA for addressing MAFLD.

## 4 Experimental Section

### Molecular Docking of OCA and AHT

The molecular structures of OCA and AHT were retrieved from the PubChem website (https://pubchem.ncbi.nlm.nih.gov/) and then optimized using ORCA 4.2.1. AutoDock Vina was employed to identify the interaction sites and docking parameters for OCA‐AHT, with visualization conducted using VMD (Visual Molecular Dynamics). The IRI analyses were performed using Multiwfn 3.8, while wave function analysis was carried out via ORCA 4.2.1 and Multiwfn software. Isosurface mapping and scatter graphs were visualized using VMD.

### Preparation of OCAHT

Obeticholic acid (OCA) and atorvastatin hemicalcium trihydrate(AHT)were dissolved in ethanol, mixed at a 2:1 molar ratio, and vortexed for 1 min. Lysine was dissolved in water as an end‐capping reagent. Water was an undesirable solvent in this preparation system. OCAHT nanocrystals were produced by quickly mixing the aqueous solution and ethanol solution via a multiple input vortex mixer (MIVM). Then, OCAHT nanocrystal powder was obtained via freeze‐drying after dialysis in ionized water with a dialysis bag (3.5 KD) for 24 h.

### Stability of OCAHT

To verify the stability of OCAHT nanocrystals in gastric and intestinal fluids, the OCAHT solution was added to simulated gastric fluid (pH 2.0), which was then placed in a 37 °C shaking bed with a constant temperature and continuous shaking at 80 rpm, and the samples were taken at 0, 1, 2, and 4 h. OCAHT nanocrystals were removed from the simulated intestinal fluid (pH 7.0) 4 h later. The samples were incubated at a constant temperature on a shaking bed. At 6, 8, 10, and 12 h, the samples were taken out. Finally, the particle size change of OCAHT nanoparticles was determined by using a Malvern Zetasizer instrument and transmission electron microscopy (Talos L 120c, Thermo Fisher).

### In Vitro Drug Release Behavior

To study the drug release behavior of OCAHT, free OCA, and AHT, the solutions of OCAHT, free OCA, and AHT were individually placed in dialysis bags with a molecular weight cutoff of 3.5 KD and dispersed in simulated body fluid (SBF) at pH 7.3. The samples were collected at 0, 0.5, 1, 2, 4, 6, 8, 12, 24, and 48 h, with an equivalent volume of fresh medium added after each collection. The released amounts of OCA and AHT in the medium were analyzed using HPLC and UV.

### Cellular Uptake

Caco‐2 cells were cultured on a polycarbonate filter in Transwell 12‐well plates for 14 days. To assess the impact of ASBT on the uptake of FITC, OCA/F, OCA‐FITC, and OCAHT‐FITC, the inhibitor sodium deoxycholate (DOCA, 200 µm) was applied to both the apical and basolateral regions of the Transwell plates for 0.5 h. Subsequently, FITC, OCA/F, OCA‐FITC, and OCAHT‐FITC were added and incubated for 2 h. Data were obtained using a flow cytometer (FongCyte, Beijing Challen Biotechnology Co., Ltd) and analyzed using the FlowJo V10 software.

To visualize the uptake of nanocrystals, the cell monolayers were treated with FITC, OCA/F, OCA‐FITC, and OCAHT‐FITC for 2 h, with a final FITC concentration of 10 µg mL^−1^. Subsequently, the monolayers were washed with PBS and stained with DAPI (5 µg mL^−1^) to label the nuclei. Next, the cell slips were transferred from the 24‐well plate to slides sealed with a tablet sealer. The slides were then examined using CLSM (Olympus SpinSR10), and the images were processed using ImageJ.

### Biodistribution of Self‐Assembled Nanocrystals Based on OCA

To study the biodistribution of OCA, AHT, and OCAHT, male Balb/c mice were randomly grouped (*n* = 3 in each group) and intragastrically administered OCA plus FITC, OCA/F, OCA‐FITC, or OCAHT‐FITC, and fasted overnight at a final OCA dose of 9.4 mg kg^−1^. The mice were sacrificed at 4 and 8 h after intragastric administration. The liver, spleen, kidney, heart, lung, and intestine were collected, and the total amount of fluorescence in the organs was measured using IVIS Spectrum (IVIS, PerkinElmer, USA).

### Detection of Liver Drug Content

This part of the study was carried out on male C57BL/6 J mice. OCA + AHT and OCAHT were administered orally at a dose of 9.4 mg kg^−1^ OCA and 12 mg kg^−1^ AHT. Liver tissues were collected at 4 and 8 h post‐administration and subsequently homogenized with methanol (in a ratio of 3 mL of methanol per gram of liver). Following centrifugation at 2500 × g for 10 min, 100 µL of the resulting supernatant was mixed with 100 µL of methanol, vortexed for 10 min, and then further centrifuged at 10 000 × g for 10 min. The resulting supernatant was subjected to analysis via HPLC.

### Preparation of the Tissue and Serum Samples

For the acute liver injury model, blood was collected from each mouse, which was then separated through centrifugation at 1000 × g for 10 min. The separated serum was subsequently stored at 4 °C for later biochemical analysis. Additionally, the liver samples were preserved in 4% paraformaldehyde to facilitate histological assessments.

For the chronic liver injury model, the blood samples were treated in the same way as the acute liver injury model. The liver of each mouse was divided into three sections, one of which was preserved in 4% paraformaldehyde for subsequent histological analysis. The remainder was stored in a −80 °C refrigerator, and one section was weighed for the detection of liver‐related indicators. Another section was used for transcriptomic analysis.

### Biochemical Assays

Serum indicators, including TC, LDL‐c, AST, and ALT, were tested using an automated biochemical analyzer (Rili3100, Rili). For liver‐related indicators, normal saline was added at a 1:9 weight‐to‐volume ratio, resulting in the addition of nine times the normal saline volume. The samples were mechanically homogenized in an ice water bath and then centrifuged at 1000 × g for 10 min, and the supernatant was collected for measurement. The lipid accumulation indexes TC, TG, and NEFA; inflammatory indexes IL‐6 and TNF‐α; and antioxidant indexes SOD, GSH‐Px, and MDA were measured following the instructions provided by the kit supplier.

### Transcriptome Sequencing Analysis

The expression levels of individual transcripts were quantified using the fragments per kilobase of exon per million mapped reads (FPKM) method for bioinformatics analysis. Differential expression analysis was performed using DEGseq with |log2 FC| ≥ 0.5 and Q value ≤ 0.001, and Gene Ontology (GO) enrichment analysis of the annotated DEGs was carried out using Phyper based on the hypergeometric test. Significance levels of terms and pathways were adjusted using the Bonferroni correction with a stringent Q value threshold.

### Immunofluorescence Staining

Immunohistochemistry (IHC) was performed with the following antibodies: anti‐PDK4 antibody (1:50, Thermo Fisher), anti‐FABP1 antibody (1:1000, Servicebo), anti‐PPARγ antibody (1:1000, Servicebo), anti‐CTSS antibody (1:200, Thermo Fisher), anti‐NLRP3 antibody (1:200, Servicebo), anti‐GSTT2 antibody (1:100, Thermo Fisher), and anti‐Nrf2 antibody (1:1000, Servicebo). The detailed experimental procedures were the same as in the previously published article.^[^ ^32^ ^]^ Immunofluorescence staining was analyzed and photographed using a microscope (DM750, Leica).

## Conflict of Interest

The authors declare no conflict of interest.

## Author Contributions

H.L., Z.B., and K.X. contributed equally to this work. H.L., Z.B., M.S., T.S., and Y.L. performed the experiments. F.C., and K.X. performed the data analysis. H.L. and M.S. performed data interpretation and drafted the manuscript. L.C., D.S., M.Z., and W.L. conceived and supervised the project and revised the manuscript.

## Supporting information

Supporting Information

## Data Availability

The data that support the findings of this study are available from the corresponding author upon reasonable request.
