# Supplementary material for: Hepatic‐Accumulated Obeticholic Acid and Atorvastatin Self‐Assembled Nanocrystals Potentiate Ameliorative Effects in Treatment of Metabolic‐Associated Fatty Liver Disease
Source: Adv Sci (Weinh). 2024 Jan 9;11(10):2308866. doi: 10.1002/advs.202308866 (PMC10933608; doi:10.1002/advs.202308866)
Supplement: Supplementary file 1 — Supporting Information [file ADVS-11-2308866-s001.pdf]

ADVANCED SCIENCE
Open Access

Supporting Information

for *Adv. Sci.*, DOI 10.1002/advs.202308866

Hepatic-Accumulated Obeticholic Acid and Atorvastatin Self-Assembled Nanocrystals Potentiate Ameliorative Effects in Treatment of Metabolic-Associated Fatty Liver Disease

Huanfen Lu, Zhenglan Ban, Kai Xiao, Madi Sun, Yongbo Liu, Fangman Chen, Tongfei Shi, Li Chen, Dan Shao, Ming Zhang* and Wei Li**

Supporting Information

Hepatic-accumulated obeticholic acid and atorvastatin self-assembled nanocrystals potentiate ameliorative effects in treatment of metabolic-associated fatty liver disease

Huanfen Lu, Zhenglan Ban, Kai Xiao, Madi Sun, Yongbo Liu, Fangman Chen, Tongfei Shi, Li Chen, Dan Shao, Ming Zhang*, and Wei Li**

H. Lu, M. Sun, T. Shi, Prof. D. Shao

School of Biomedical Sciences and Engineering, South China University of Technology, Guangzhou, Guangdong, 511442, China

E-mail: stanauagate@outlook.com

College of Chinese Medicinal Materials, Jilin Agricultural University, 130118, Changchun, China

E-mail: liwei7727@126.com

H. Lu, Z. Ban, M. Sun, K. Xiao, T. Shi, F. Chen, Prof. D. Shao

National Engineering Research Center for Tissue Restoration and Reconstruction, South China

University of Technology, Guangzhou, Guangdong, 510006, China

Z. Ban, K. Xiao, Prof. D. Shao

School of Medicine, South China University of Technology, Guangzhou, Guangdong 510006, China

Y. Liu, Prof. W. Li

Prof. L. Chen, Prof. M. Zhang

College of Medicine, Jilin University, 130021, Changchun, China

E-mail: zhangming99@jlu.edu.cn

Experimental Section

Characterization of nanoparticles

Dynamic light scattering (DLS) and zeta potential measurements of the OCAHT nanocrystals were performed to obtain the size distribution and charge characteristics via a Malvern Zetasizer Nano-ZS (DLS; Malvern Instrument, UK). The morphology of the OCAHT NPs were imaged by scanning electron microscopy (Q25, Thermo Fisher). X-ray diffraction (XRD) patterns were collected to analyze the morphology of the nanocrystals (SE, Rigaku SmartLab) with 2θ values ranging from 5° to 90°. To verify the successful assembly of OCA and AHT, a Fourier transform infrared spectrometer (Nicolet iS50, Thermo Fisher) was utilized to detect and analyze OCA, AHT and OCAHT.

Cells and animals

Caco-2 cells, HepG2 cells and RAW 264.7 cells were cultured in DMEM medium (Gibco, California, USA) with 10% (v/v) FBS fetal bovine serum (Gibco, Vienna, Austria) and a 1% penicillin–streptomycin mixture. The cells were cultured at 37 °C in an incubator with 5% CO_2.

Male C57BL/6J mice (5-7 weeks) were purchased from Hunan SJA Laboratory Animal Limited Company and acclimatized. The whole animal experiments were approved by the Institutional Animal Care and Use Committee of South China University of Technology (Guangzhou, China). The mice were used in formal experiments after they were put through a week of quarantine at the animal center. The living space of the mice was kept at a constant temperature of 23 ± 2 °C, 60-70% humidity, and a 12 h light/dark cycle.

Preparation of OCAHT-FITC

OCA and FITC were injected into anhydrous DMSO at a molar ratio of 1:1, followed by heating at 50 °C for 24 h to produce OCA-FITC. Subsequently, the self-assembly of OCA-FITC and AHT was carried out to obtain OCAHT-FITC although the synthesis method of OCAHT.

Animal treatment

For the acute liver injury model, male C57BL/6J wild-type mice (6–8 weeks) were

randomly grouped (n = 4 in each group). After fasting for 16 h, APAP (Sigma-Aldrich) was given at a dose of 200 mg/kg by a single i.p. injection. to induce AILI. 12 h before, 1 h after and 12 h after APAP modeling, intragastric administration with free OCA, free AHT, combined OCA and AHT, or OCAHT. In the treatment groups without predosing, medication was administered at 1 and 12 h after APAP-challenging, following the same procedures as described above.

For the chronic liver injury model, male C57BL/6J wild-type mice (6–8 weeks) were randomly divided into six groups (n = 4 in each group): one normal diet group (control group) and five high-fat diet groups. The high-fat diet was made with 35% fat, 26% protein and 26% carbohydrate (H10060, Huafukang Biology, Beijing), and the mice were also given 15% fructose water in the HFD group. The drug was administered after 8 weeks of feeding. The control group was treated daily with saline via oral gavage for 8 weeks. The five high-fat diet groups were orally treated with saline (Model group), free OCA, free AHT, combined OCA and AHT, and OCAHT for 8 weeks. All formulation groups were treated with 9.4 mg/kg OCA and 12 mg/kg AHT. The body weights of the mice were recorded every week over the course of the study.

Drug safety evaluation at the animal level

Male C57BL/6J mice were orally administered OCA, AHT, OCA + AHT, and OCAHT continuously for one week at a dose of 9.4 mg/kg for OCA and 12 mg/kg for AHT. Following the experiment, we detected mouse serum AST and ALT and stained the liver with H&E.

Histological assessment

For the acute liver injury model, after being submerged in 4% paraformaldehyde, the harvested tissues were embedded in paraffin blocks and sliced for H&E staining.

For the chronic liver injury model, the liver sections were stained with Oil Red O and counterstained with hematoxylin to evaluate hepatic lipid accumulation after H&E staining. The heart, spleen, lung and kidney were fixed in 4% paraformaldehyde for H&E staining to evaluate the biocompatibility of drugs.

Hepatocyte steatosis and oxidative stress therapy

HepG2 cells were loaded with FFA (oleic acid: palmitic acid = 2:1) for 8 h to

mimic hepatic steatosis. HepG2 cells were exposed to FFA with OCA, AHT, OCA + AHT and OCAHT pretreatment for 16 h. The cells were stained with Nile Red, and then fluorescence images were taken.

HepG2 cells were loaded with FFA (oleic acid: palmitic acid = 2:1) for 24 h to mimic oxidative stress in hepatocytes, and OCA, AHT, OCA + AHT and OCAHT were added together with FFA. The cells were stained with DCFH-DA, and then fluorescence images were taken.

Cellular inflammation therapy

RAW264.7 cells were seeded into 24-well plates at a density of 1×10^6 cells/well. When reaching 90% confluency, RAW264.7 cells were incubated with progressive concentrations of OCA, AHT, OCA + AHT and OCAHT or vehicle (PBS) for 30 min before the addition of 10 ng/mL LPS. Then, the cells were further cultured for an additional 24 h prior to cell harvesting for TNF-α detection.

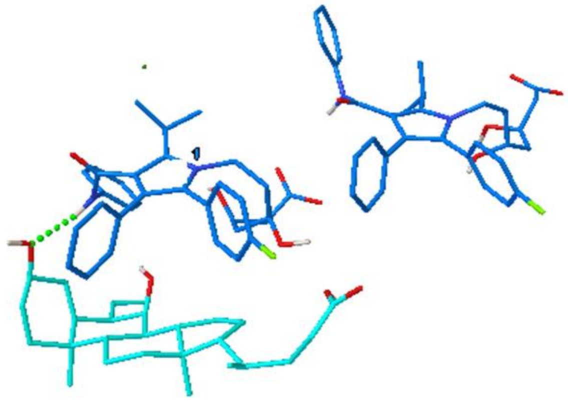

Figure S1. **The binding energy between OCA and AHT is determined by molecular dynamics simulations.** Molecular dynamics simulations of OCA (right) and AHT (left), and the results showed that their binding energy was -4.5 kJ/mol.

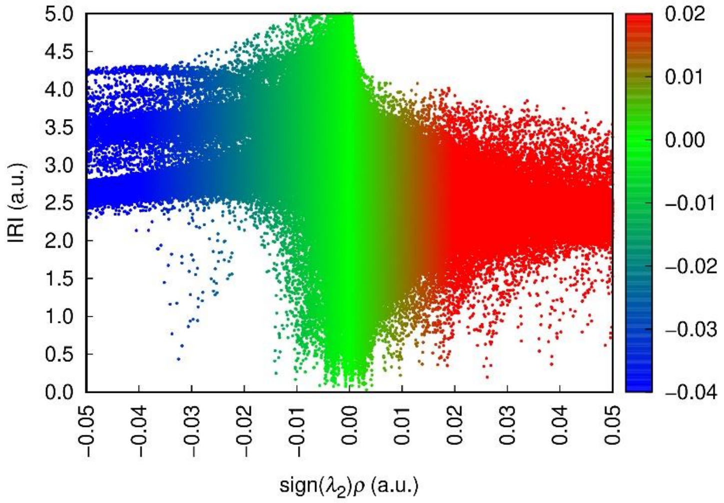

Figure S2. **IRI standard coloring.** Standard coloring method of sign(λ2)ρ on IRI isosurfaces.

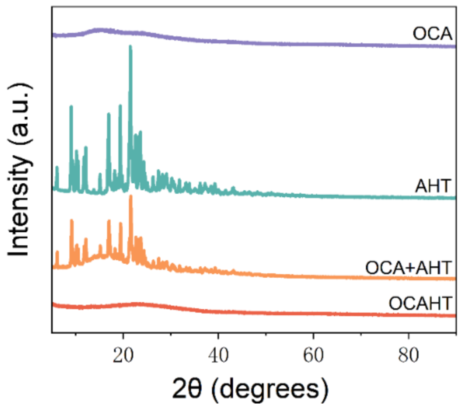

Figure S3. **Amorphous nanocrystal of OCAHT is analyzed by XRD.** XRD of the OCAHT nanocrystals compared to the OCA, AHT and their physical mixture.

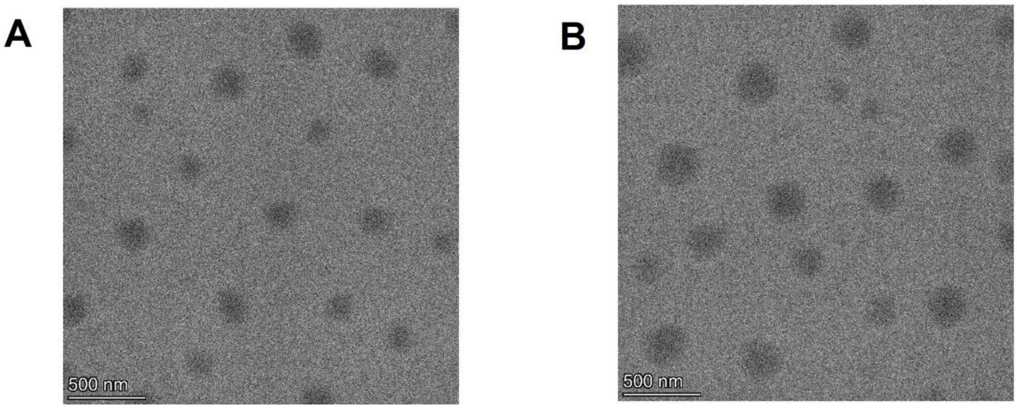

Figure S4. **TEM images confirm the stability of OCAHT in SGF and SIF.** The morphology of OCAHT after incubation with simulated gastric fluid for 4 h (A) and simulated intestinal fluid for 4 h (B) was characterized by TEM. Scale bar: 500 nm.

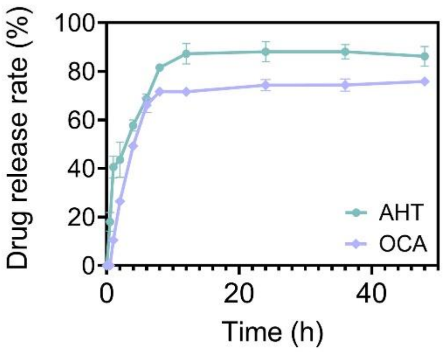

Figure S5. **Free OCA and AHT suspensions release rapidly in SBF.** Cumulative release curve of free OCA and AHT suspension in SBF for 48 h. Data are given as mean ± SD (n = 3).

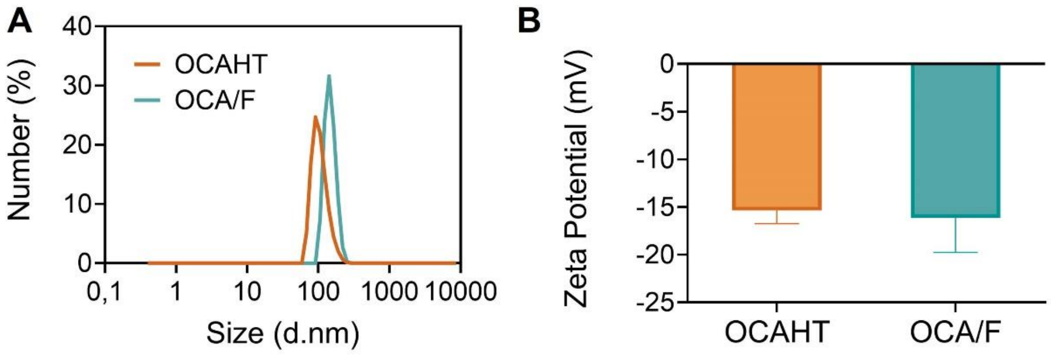

Figure S6. **The particle size and zeta potential of OCA/F and OCAHT are similar.** (A) Size distribution of OCAHT and OCA/F. (B) Zeta potential of OCAHT and OCA/F.

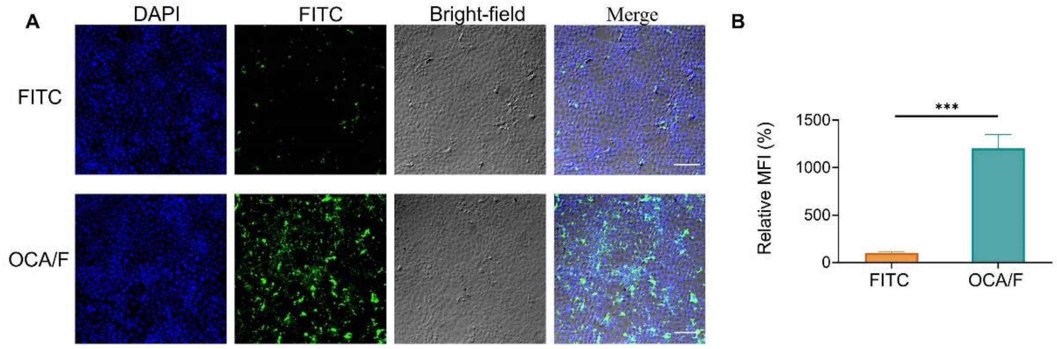

Figure S7. **The uptake of OCA/F by Caco-2 cells is much higher than that of FITC.** (A) CLSM images of Caco-2 cell monolayers incubated with OCA/F and FITC for 2 h. Nuclei were stained with DAPI (blue). Scale bar: 100 µm. (B) Quantitative determination of fluorescence amounts between the two groups. Data are expressed as mean ± SD (n = 4), ***P < 0.001.

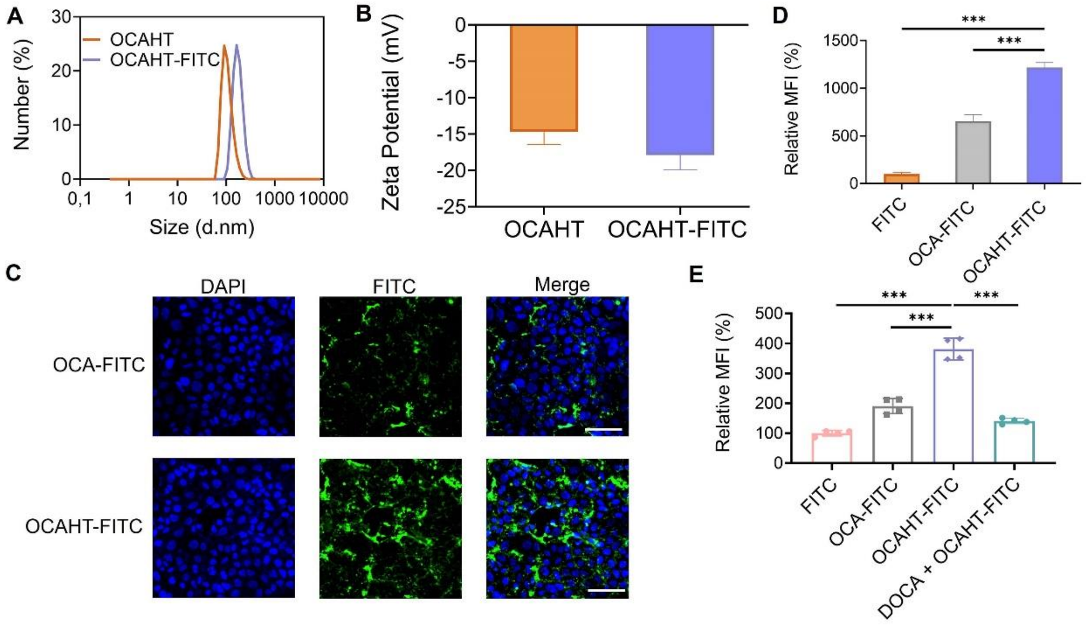

Figure S8. **The uptake of OCAHT-FITC by Caco-2 cells is much higher than that of OCA-FITC.** (A) Size distribution of OCAHT and OCAHT-FITC. (B) Zeta potential of OCAHT and OCAHT-FITC. (C) CLSM images of Caco-2 cell monolayers incubated with OCAHT-FITC and OCA-FITC for 2 h. Nuclei were stained with DAPI (blue). Scale bar: 50 μm. (D) Quantitative determination of fluorescence between the three groups. (E) Quantitative detection of cellular internalization in various groups. Data are expressed as mean ± SD (n = 4), ***P < 0.001.

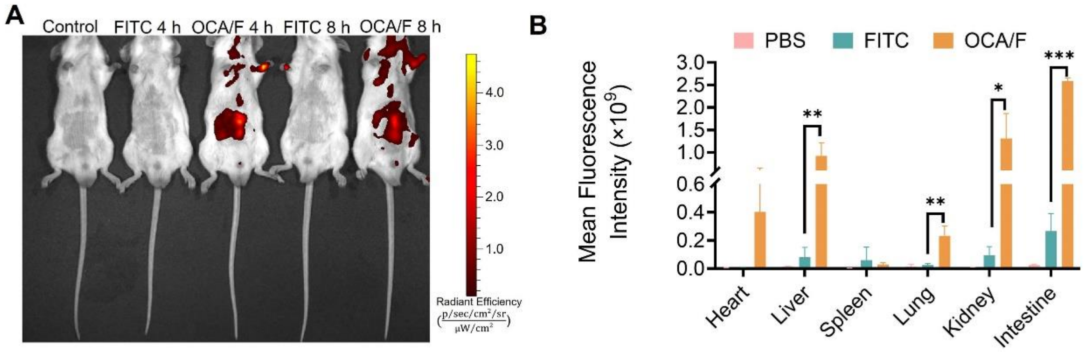

Figure S9. **The liver accumulation of OCA/F is much higher than that of FITC.** (A) Whole-body fluorescence images of mice at 4 and 8 h after mice were treated with OCA + FITC or OCA/F via oral administration. (B) Mean fluorescence intensity of organs at 4 h after mice were treated with FITC and OCA/F via oral administration. Data are expressed as mean ± SD (n = 3), $^{*}P < 0.05$, $^{**}P < 0.01$, $^{***}p < 0.001$.

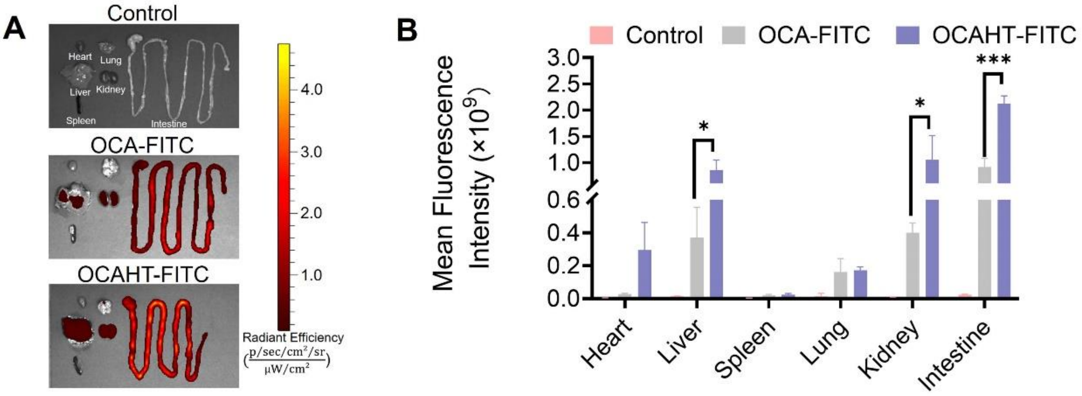

Figure S10. **The liver accumulation of OCAHT-FITC is much higher than that of OCA-FITC.** (A) Fluorescence images of main organs at 4 h after mice orally received OCAHT-FITC or OCA-FITC. (B) Mean fluorescence intensity of organs at 4 h after mice were treated with OCA-FITC and OCAHT-FITC via oral administration. Data are expressed as mean ± SD (n = 3), *P < 0.05, **P < 0.01, ***p < 0.001.

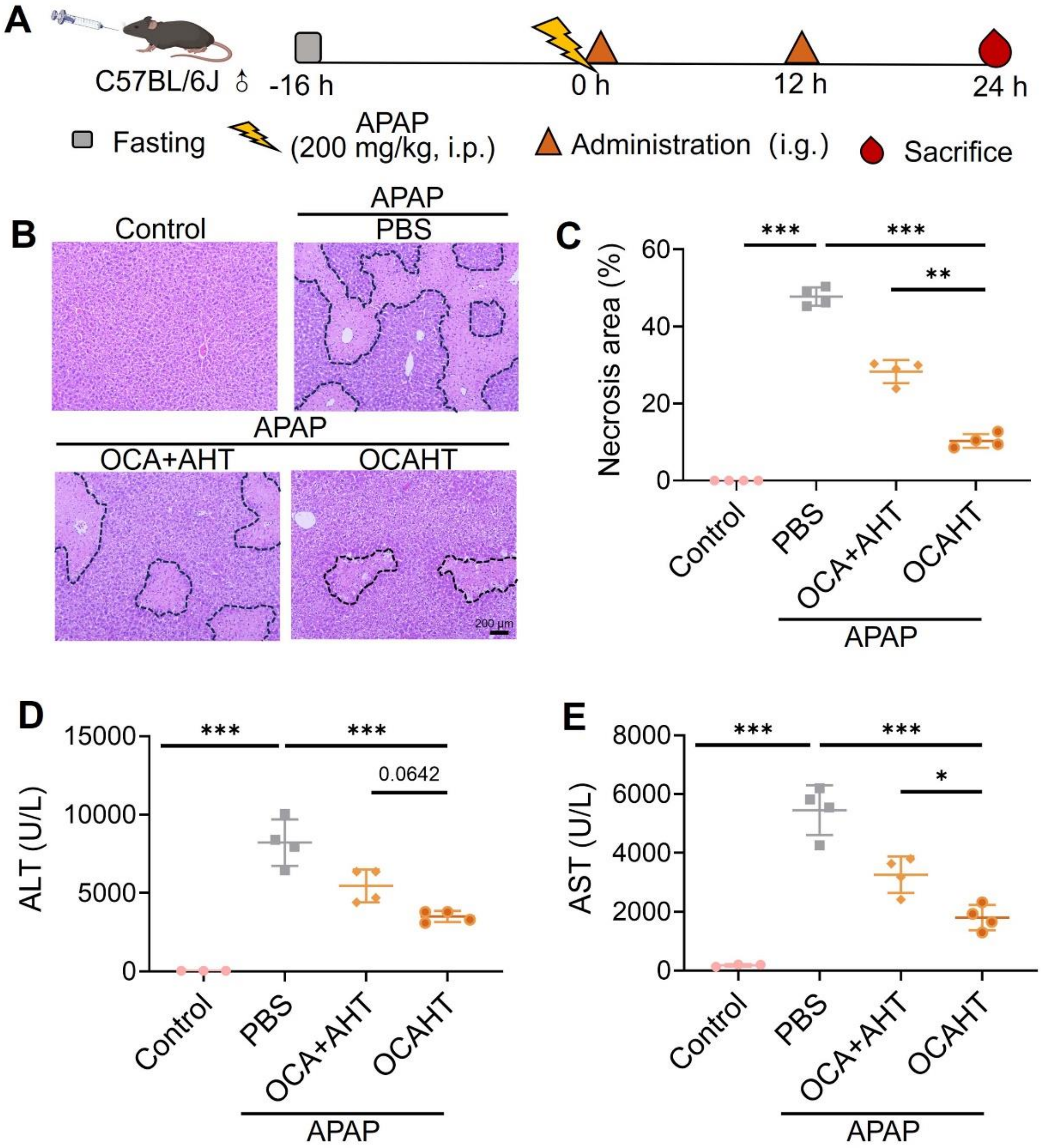

Figure S11. **OCAHT protects against acute liver damage and inflammation without pre-medication.** (A) Schematic illustration of the development of APAP-challenged mice and the therapeutic effect of OCAHT against ALI. (B) Representative H&E staining of liver. Scale bar: 200 μm. (C) The level of necrosis area. (D-E) The levels of ALT and AST in serum at 24 h after APAP-challenged. Data are expressed as mean ± SD (n = 4), *P < 0.05, **P < 0.01, ***p < 0.001.

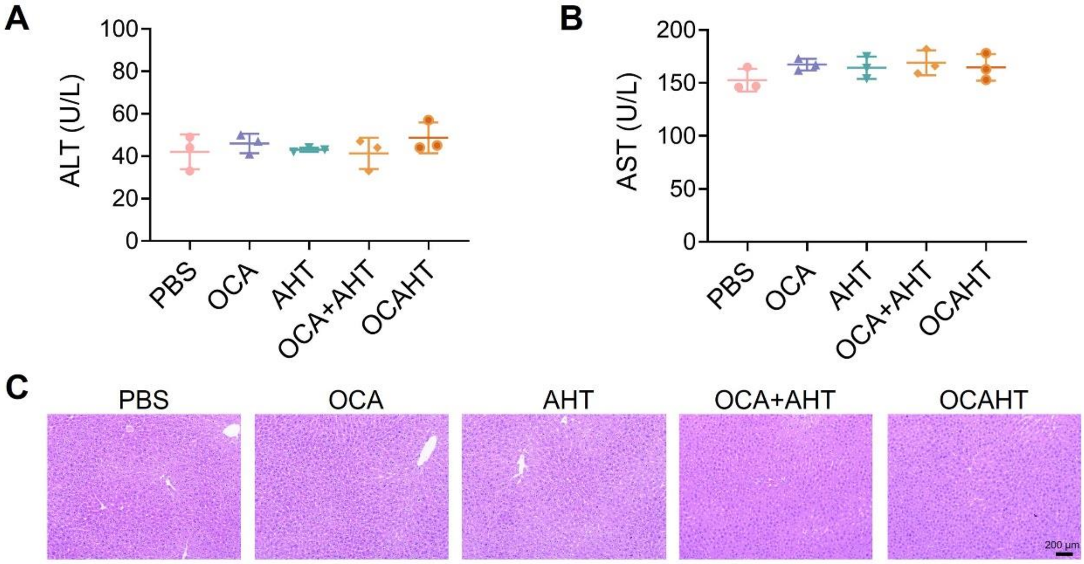

Figure S12. **OCA, AHT and OCAHT have no effect on liver function in mice.** (A-B) ALT and AST levels of healthy mice liver after treatment with OCA, AHT, OCA + AHT and OCAHT for one week of continuous intragastric administration. Data are expressed as mean ± SD (n = 3). (B) Representative H&E staining of liver. Scale bar: 200 μm.

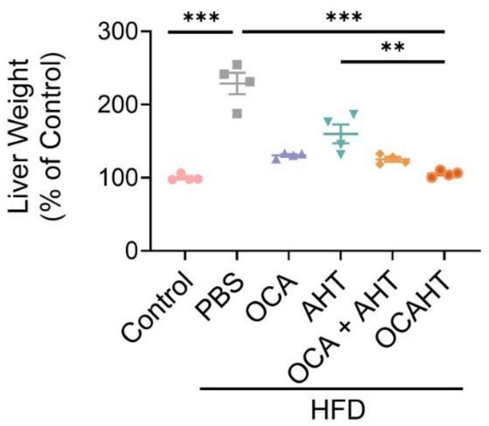

Figure S13. **OCAHT reduces liver weight in HFD mice.** Liver weight of HFD-challenged mice after treatment. Data are expressed as mean ± SD (n = 4), *P < 0.05, **P < 0.01, ***p < 0.001.

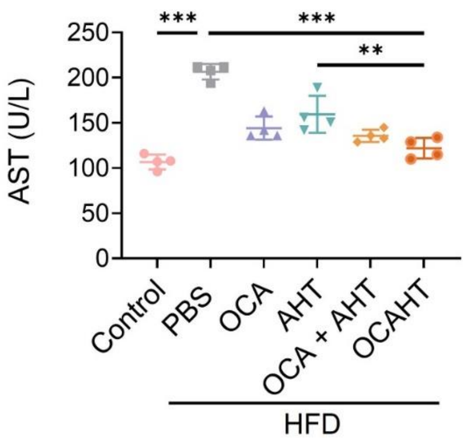

Figure S14. **OCAHT alleviates liver damage in HFD mice.** The levels of AST in serum after treatment. Data are expressed as mean ± SD (n = 4), *P < 0.05, **P < 0.01, ***p < 0.001.

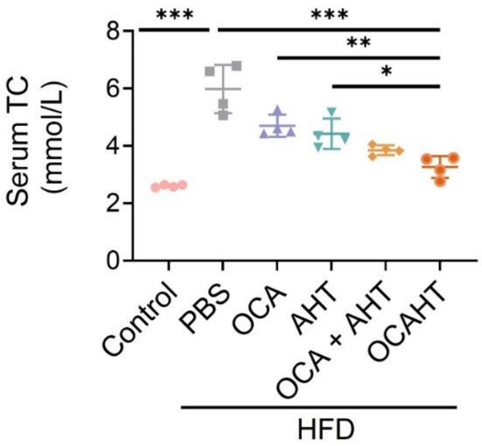

Figure S15. **OCAHT reduces lipid accumulation in HFD mice.** The levels of TC in serum after treatment after treatment. Data are expressed as mean ± SD (n = 4), *P < 0.05, **P < 0.01, ***p < 0.001.

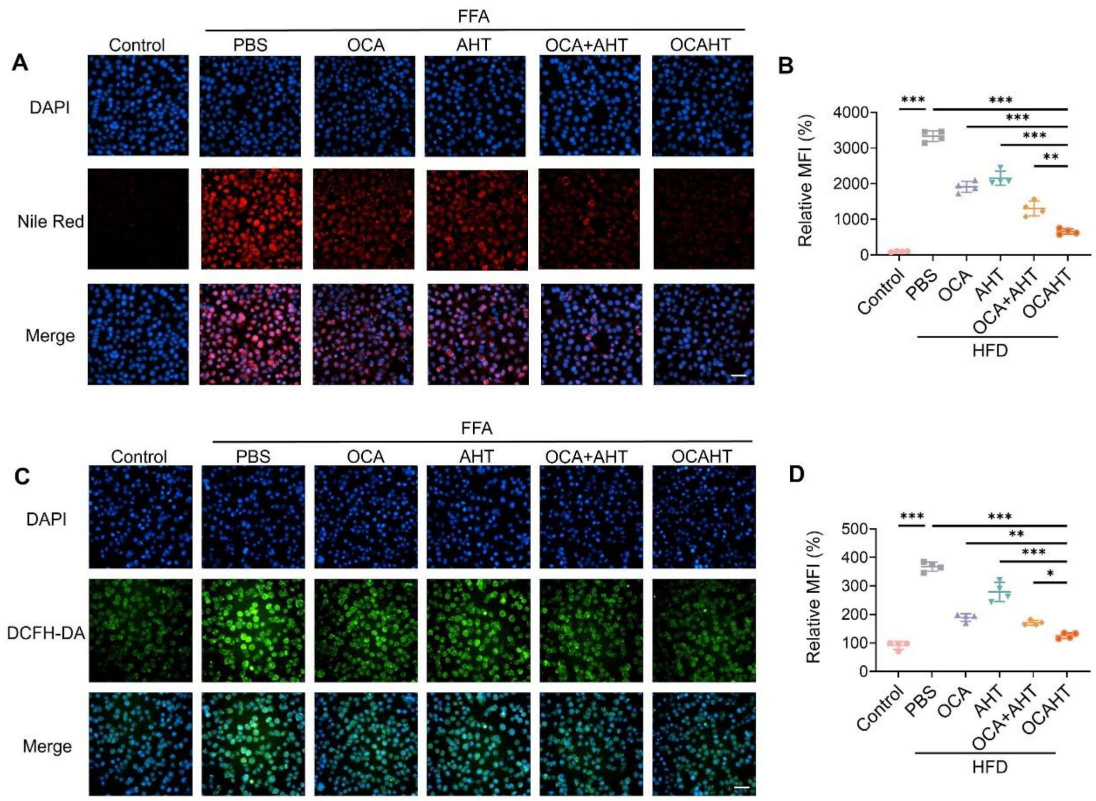

Figure S16. **OCAHT reduces lipid accumulation and improves oxidative stress in HepG2 cells.** (A) Intracellular lipid accumulation was measured by Nile Red staining. Nuclei were stained with DAPI (blue). Scale bar: 50 µm. (B) Quantitative determination of fluorescence amounts of lipid accumulation. (C) Intracellular ROS levels were measured using the fluorescent probe DCFH-DA. Nuclei were stained with DAPI (blue). Scale bar: 50 µm. (D) Quantitative determination of fluorescence amounts of ROS. Data are expressed as mean ± SD (n = 4), *P < 0.05, **P < 0.01, ***p < 0.001.

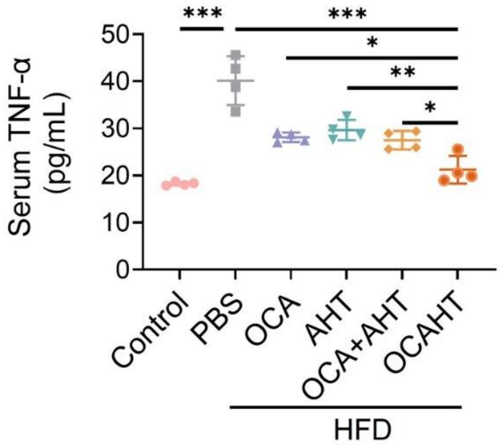

Figure S17. **OCAHT mitigates inflammation in HFD mice.** The levels of TNF-α in serum after treatment. Data are expressed as mean ± SD (n = 4), *P < 0.05, **P < 0.01, ***p < 0.001.

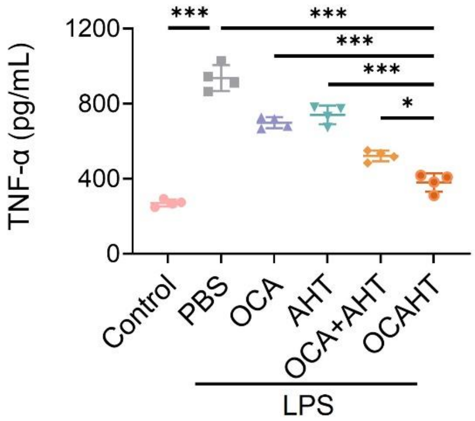

Figure S18. **OCAHT reduces pro-inflammatory cytokine in RAW264.7 cell.** The levels of TNF-α in RAW264.7 cell after treatment. Data are expressed as mean ± SD (n = 4), *P < 0.05, **P < 0.01, ***p < 0.001.

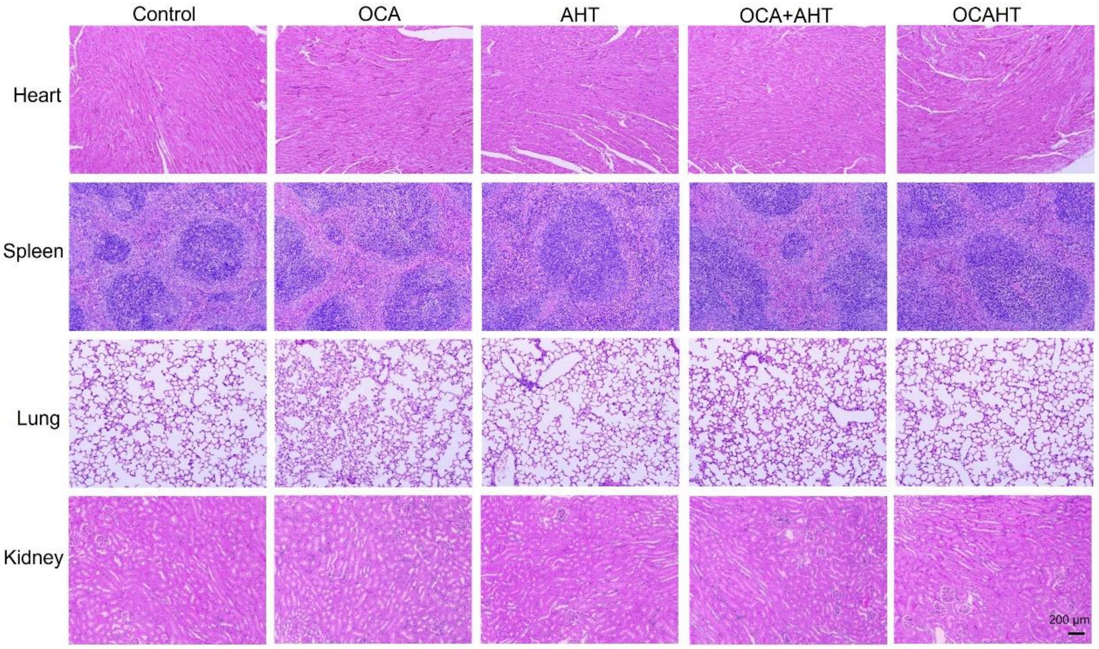

Figure S19. **OCAHT has good biosafety.** H&E staining images of major organs, including the heart, spleen, lung, kidney. Scale bars: 200 μm.

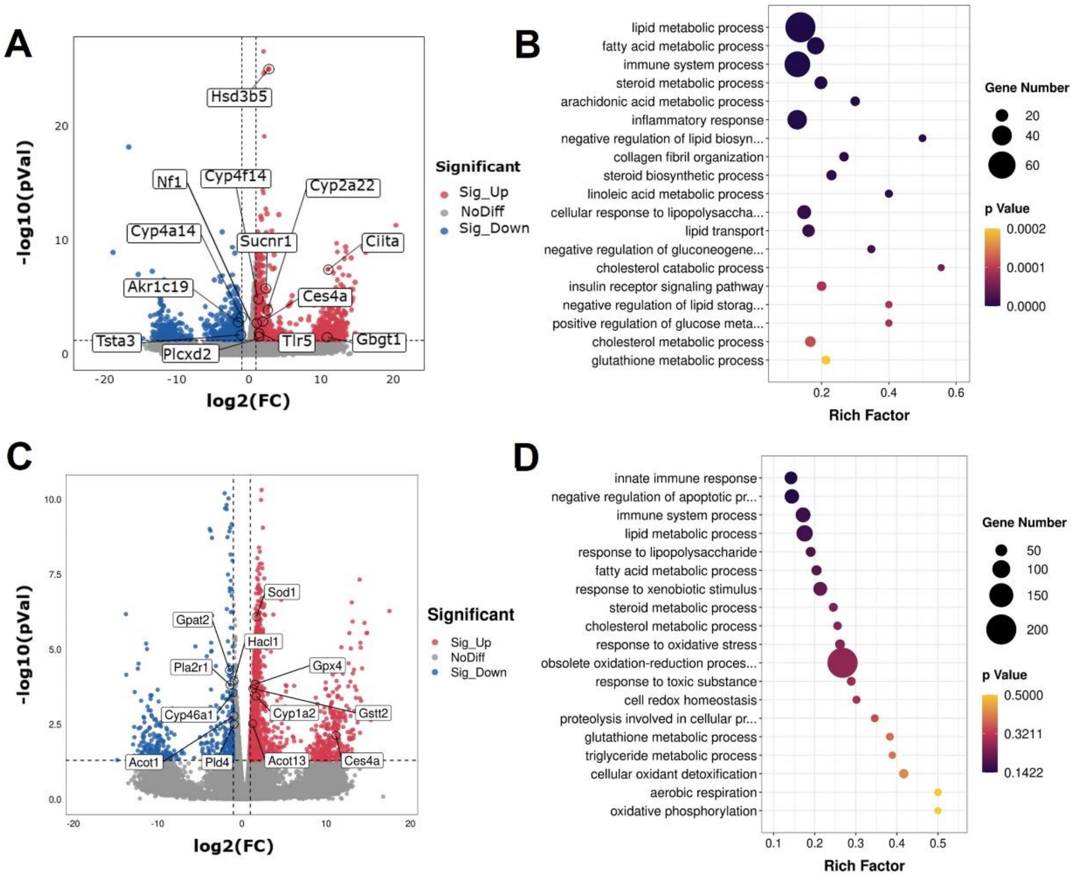

Figure S20. **Decipher the potential role of OCAHT in MAFLD treatment.** Volcano map of differentially expressed genes in mouse liver between HFD vs. Control (A) and OCAHT vs. OCA + AHT (C) after eight weeks of treatment. The GO functional enrichment analysis of DEGs between HFD vs. Control (B) and OCAHT vs. OCA + AHT (D) after eight weeks of treatment.

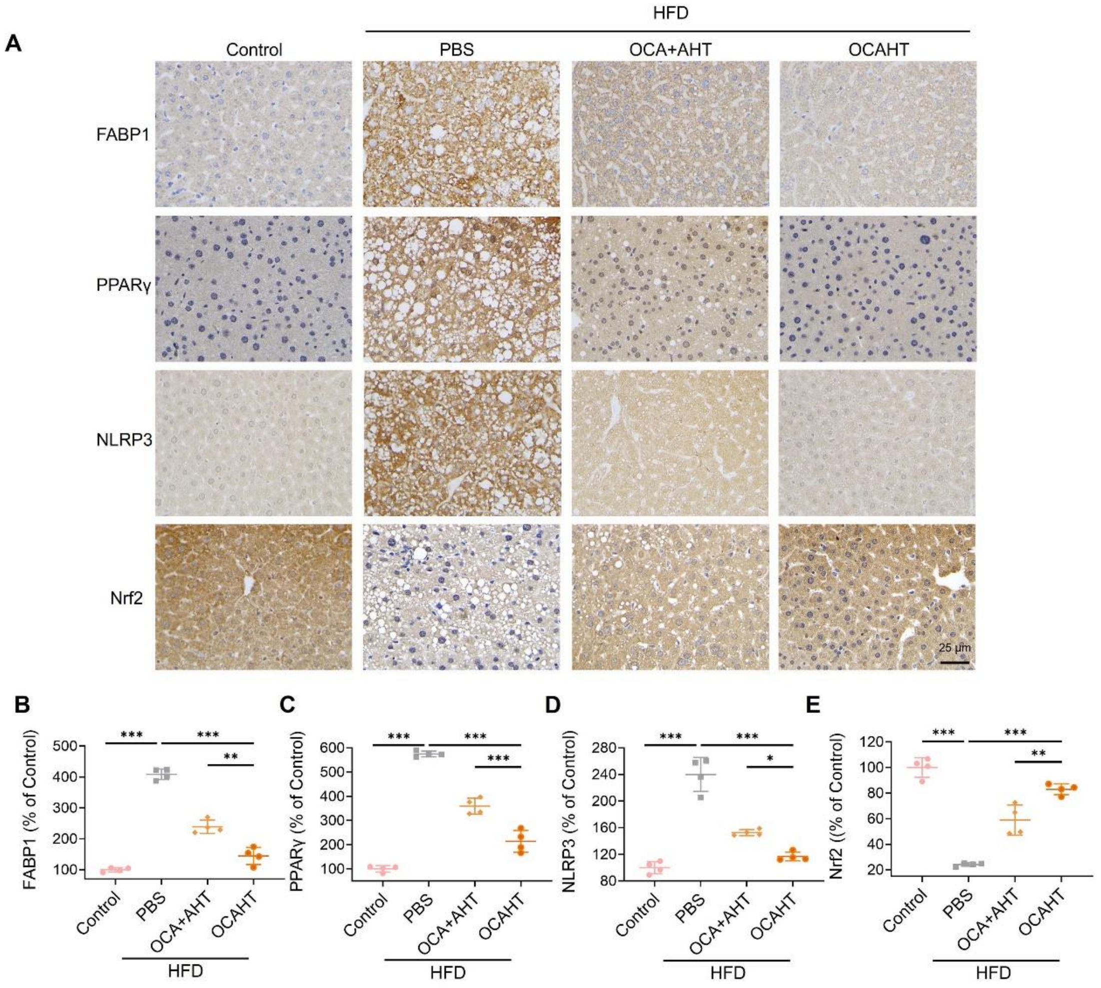

Figure S21. OCAHT orchestrates the expression of MAFLD-related proteins. (A) Representative immunohistochemical staining of liver for FABP1, PPARγ, NLRP3, and Nrf2 after treatment. Scale bar: 25 μm. Quantitative analysis results of the levels of FABP1 (B), PPARγ (C), NLRP3 (D), and Nrf2 (E) for the images in A via ImageJ. Data are expressed as mean ± SD (n = 4), *P < 0.05, **P < 0.01, ***p < 0.001.
